# Mutations Related to Antibiotics Resistance in *Helicobacter pylori* Clinical Isolates from Bangladesh

**DOI:** 10.3390/antibiotics12020279

**Published:** 2023-01-31

**Authors:** Kartika Afrida Fauzia, Hafeza Aftab, Evariste Tshibangu-Kabamba, Ricky Indra Alfaray, Batsaikhan Saruuljavkhlan, Alain Cimuanga-Mukanya, Takashi Matsumoto, Phawinee Subsomwong, Junko Akada, Muhammad Miftahussurur, Yoshio Yamaoka

**Affiliations:** 1 Department of Environmental and Preventive Medicine, Faculty of Medicine, Oita University, Yufu 879-5593, Japan; 2 Department of Public Health and Preventive Medicine, Faculty of Medicine, Universitas Airlangga, Surabaya 60115, Indonesia; 3 Helicobacter pylori and Microbiota Study Group, Institute of Tropical Disease, Universitas Airlangga, Surabaya 60115, Indonesia; 4 Department of Gastroenterology, Dhaka Medical College and Hospital, Dhaka 1000, Bangladesh; 5 Research Center for Infectious Sciences, Department of Parasitology, Graduate School of Medicine, Osaka City University, Osaka 545-8585, Japan; 6 Department of Internal Medicine, Faculty of Medicine, Pharmacy and Public Health, University of Mbujimayi, Mbujimayi 225-80, Democratic Republic of the Congo; 7 Department of Microbiology and Immunology, Hirosaki University Graduate School of Medicine, Hirosaki 036-8562, Japan; 8 Division of Gastroentero-Hepatology, Department of Internal Medicine, Faculty of Medicine-Dr. Soetomo Teaching Hospital, Universitas Airlangga, Surabaya 60131, Indonesia; 9 Department of Medicine, Gastroenterology and Hepatology Section, Baylor College of Medicine, Houston, TX 77030, USA; 10 Research Center for Global and Local Infectious Diseases, Oita University, Yufu 879-5593, Japan; 11 Borneo Medical and Health Research Centre, University Malaysia Sabah, Kota Kinabalu, Sabah 88400, Malaysia

**Keywords:** *Helicobacter pylori*, antimicrobial resistance, mutations, Bangladesh, infectious disease

## Abstract

Current management of gastric inflammation involves the eradication of *Helicobacter pylori*. However, the effectiveness of commonly used antibiotics against *H. pylori* infection has decreased due to antibiotic resistance. Phenotypic-based diagnostics are laborious and finding the cause of resistance can be difficult. Therefore, early detection and understanding of the underlying mechanism of this resistance are necessary. This study evaluated the mutations in the genes related to the Antimicrobial Resistance (AMR) of the clinical isolates from Bangladeshi subjects. Whole-genome sequencing was performed on 56 isolates and the genes (such as *pbp1a*, *rdxA*, *ribF*, *fur*, *gyrA*, *gyrB*, *23S rRNA,* and *infB*) were extracted. The reads were assembled, and the SNPs were extracted by the latest pipeline for antibiotic mutation analysis, ARIBA. The mutations and the association with the antibiotic phenotypes were evaluated using Fisher’s exact test. In this study, the clarithromycin resistance rate was high, 39.3% (22/56), with the median MIC 24 mg/L ranging from 2 to 128 mg/L. The mutation of A2147G was significantly associated with resistance (*p* = 0.000018) but not in locus A2146G (*p* = 0.056). Levofloxacin also posed a high resistance. We observed that the mutation of D91N (but not D91Y) (*p* = 0.002) and N87K (*p* = 0.002) of *gyrA* was associated with levofloxacin resistance. Mutations in locus A343V (*p* = 0.041) of *gyrB* also showed a significant association. Meanwhile, in the *pbp1a* gene, several mutations might explain the resistance; they were G594fs (*p* = 0.036), K306R (*p* = 0.036), N562Y (*p* = 0.0006), and V45I (*p* = 0.018). The prevalence of metronidazole was exceptionally high (96.4%), and numerous mutations occurred in *rdxA* genes, including the truncation of genes. These results imply that the mutation in genes encoding the target protein of antibiotics remains the critical resistance mechanism in *H. pylori*.

## 1. Introduction

Due to widespread antimicrobial resistance, the first-line treatment for *Helicobacter pylori* (*H. pylori*) infection has a decreasing cure rate [1,2]. In contrast, a chronic *H. pylori* infection can lead to the development of gastroduodenal diseases such as stomach cancer and peptic ulcer disease [3]. A recent agreement on *H. pylori* treatment strongly advocated modifying the treatment based on the findings of antimicrobial susceptibility testing to address the issue [4]. It hoped to improve eradication rates and lower antibiotic resistance levels globally, but some studies still found eradication failures [5]. These findings imply a knowledge gap in the mechanisms underlying antibiotic resistance. Understanding the factors that lead to antibiotic resistance can help to eradicate and manage *H. pylori* infections effectively.

Clarithromycin, amoxicillin, and proton pump inhibitors have been the initial regimens for eradicating *H. pylori*. The macrolide clarithromycin inhibits the function of 23S rRNA in protein synthesis [6]. Various global investigations have implicated point mutations in the 23S rRNA gene as the source of the development of resistance, with mutation types varying at loci 2146, 2147, and 2182 [6,7,8,9]. Other genes, including *infB*, have also been linked to the emergence of resistance [10]. Meanwhile, it has been discovered that mutations within the penicillin-binding protein gene family are a significant factor in amoxicillin resistance [11].

Significant prospects for alternate regimens include metronidazole and levofloxacin. However, resistance to levofloxacin and metronidazole increasingly continues to be an issue [2]. As well as mutations in the *gyrB* gene involved in levofloxacin resistance, mutations in the *gyrA* gene primarily occur at loci N87 and D91 [12]. As for metronidazole resistance, numerous potential genes, including *rdxA* and *frxA*, have been discovered. Now, investigations indicate that additional genes, including *ribF*, *mdaB,* and *omp11*, also play a role [7].

Compared to other bacterial species, the *H. pylori* genome is highly diverse [13,14]. Genome diversities also affect genetic material associated with antibiotic resistance, as some regions have unique properties at the gene or single nucleotide polymorphism (SNP) level. According to multiple investigations, there are still resistant bacteria for which no mutations are observed [6]. Therefore, a population must comprehensively examine the SNP level’s resistance mechanism.

This study attempted to examine known-variant and novel-variant genes involved in antibiotic resistance from Next Generation Sequencing data. Clinical *H. pylori* isolates from Bangladesh have been checked for susceptibility to amoxicillin, clarithromycin, levofloxacin, and metronidazole [15]. Whole genome analysis was then performed to understand further the mechanism of *H. pylori*’s antibiotic resistance.

## 2. Results

### 2.1. Genome Relatedness and Antibiotic Resistance

**Figure 1:**
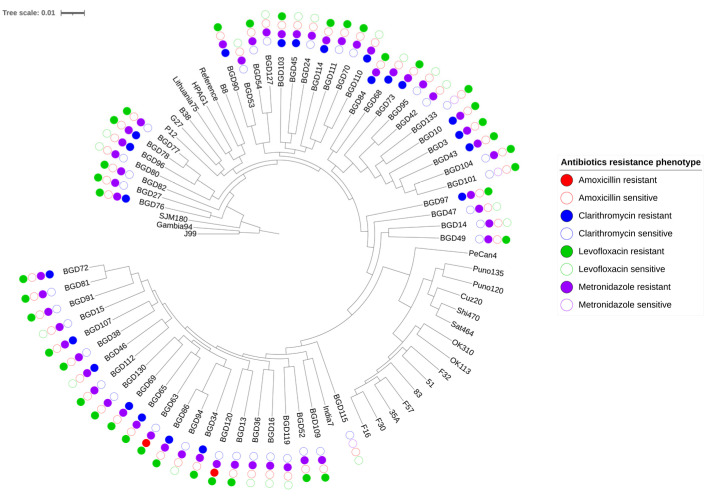
Phylogenetic tree of clinical *H. pylori* isolated from Bangladesh. Phylogenetic tree of Bangladesh clinical isolates was constructed based on the maximum likelihood method. The resistance phenotypes of antibiotics were also shown in filled color, as mentioned in the legend.

Incorporating genome alignment of Bangladeshi isolates and reference isolates whose populations had been previously identified, the maximum likelihood tree was constructed. In accordance with a previous report [16], Bangladesh isolates clustered with hpAsia2 and hpEurope, which can be distinguished from the tree (Figure 1). Levofloxacin and metronidazole resistance are distributed comparably among hpAsia2 (levofloxacin 16/23, metronidazole 22/23) and hpEurope (levofloxacin 21/33, metronidazole 32/33) Multidrug resistance was observed in 78.6% (44/56) isolates, while metronidazole resistance was observed in only 12.6% (12/56) isolates. In addition, we found that 19 of 22 clarithromycin-resistant bacteria and all amoxicillin-resistant bacteria shared resistance to levofloxacin.

### 2.2. Mutations Related to Resistance to Levofloxacin

**Figure 2:**
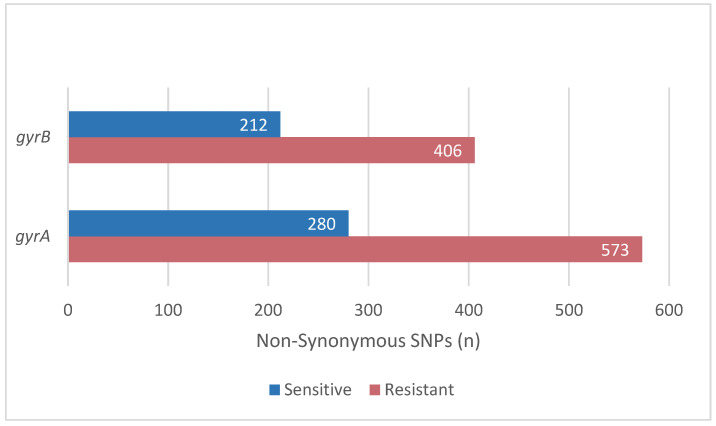
The SNPs obtained after the alignment to the *gyrA* and *gyrB* genes of *H. pylori* strain 26695.

*gyrA* and *gyrB* are the genes encoding the protein target of levofloxacin. Among the 56 genomes we evaluated, the total SNPs were 618 for *gyrA* and 853 for *gyrB* (Figure 2). The total number of mutations in the resistance isolates is higher than sensitive.

We observed that only mutations in the loci 87 and 91 of *gyrA* showed significant association with levofloxacin resistance, and none of these mutants were found in the susceptible isolates (Table 1). Only *one* strain had a mutation in both loci, and the MIC was 64 mg/L. About 87.5% of mutations in *gyrB* are complemented with the mutation in *gyrA,* while only one resistant strain possessed mutation A343V on *gyrB* but not on *gyrA.* Based on the mutations of these loci, the concordance between the genotype and phenotype was 89.2% (33/37).

A tendency of mutation N87K of the resistant isolates in the *hpEurope* cluster was observed (8/13) (Supplementary Table S1). The mutation of D91N was also more frequent in hpEurope; implying the possible influence of population type in the resistance.

### 2.3. Mutations Related to Resistance to Amoxicillin

To find the genetic variant that appeared in amoxicillin resistance, we evaluated four genes: *pbp1a. pbp2*, *pbp3*, and *pbp4* (Table 2). The substitution from Asparagine to Tyrosine in locus 562 of *pbp1a* was found in both resistant isolates but not in the susceptible isolates. The other mutation was found in only one strain each. Based on the identification through locus 562 in *pbp1a*, the agreement between the genotypic and phenotypic approaches was 100%.

### 2.4. Mutations Related to Resistance to Metronidazole

Several genes reportedly contribute to metronidazole resistance. In this study, six genes were evaluated and the association analysis was performed (Table 3). Isolates possessing frameshift mutations in various loci were reported the resistance phenotype (Supplementary Table S2). The eight loci of a frameshift mutation in *the rdxA* gene were found in 47 isolates (83.3%).

**Figure 3:**
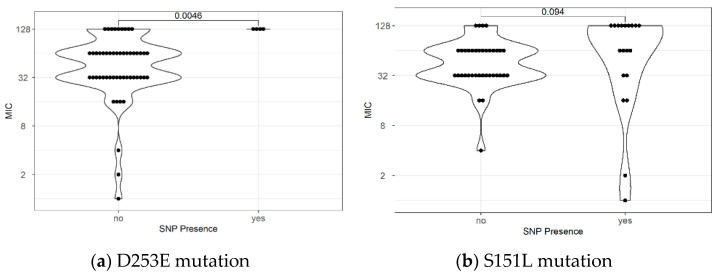
Comparison of metronidazole MIC in the presence and absence of mutations: All isolates possessing D253E mutation showed a high level of resistance (MIC 128 mg/L) (**a**). Meanwhile, the majority of high-level resistance possessed the S151L mutations (**b**).

In the *ribF* gene, the total number of variants possessed by all isolates is higher than the genes in both resistant and susceptible isolates. An average of 11.6 SNPs were present in each resistant strain, while 12.8 SNPs were present in susceptible isolates. Significant associations between the metronidazole susceptibility and the SNPs in the *ribF* gene were also observed as a substitution of aspartic acid to glutamic acid in locus 253 (D253E) and a substitution of Isoleucine to Leucine (S151L). Isolates possessing a substitution in locus D253E showed an MIC value of 128 mg/L, significantly higher compared to isolates without the mutation (Figure 3a).

The protein physical and chemical parameters analysis of proteins Fur and RibF from the wild-type strain compared to the mutant showed a difference in the instability index (Supplementary Table S3). In the case of the Fur protein, the wild-type had a lower instability index, meaning this protein was more stable than the mutant (see the mutation position in Supplementary Figure S1A). As for the RibF protein, the wild-type and the mutant D253E showed similar values for all parameters. The wild-type had a lower aliphatic index, meaning this protein had slightly lower thermal stability than mutant RibF S151L (see the mutation position in Supplementary Figure S1B,C).

### 2.5. Mutations Related to Resistance to Clarithromycin

For the clarithromycin resistance, we evaluated 23S rRNA and *infB.* The resistance to clarithromycin is more prevalent in the HpEurope (48.5%) than in hpAsia2 (26.5%). A mutation in A2147G was also more frequent in the hpEurope isolates than the others. Among resistant isolates with A2147G mutation, 90% belonged to hpEurope.

Among 22 isolates resistant to clarithromycin, 14 possessed a mutation in loci 2146 and 2147. Interestingly, none of them had a mutation in both loci (Table 4). This indicated that, based on the mutation of 2146 and 2147 only, the agreement between genotype and phenotype was 63.6%.

**Figure 4:**
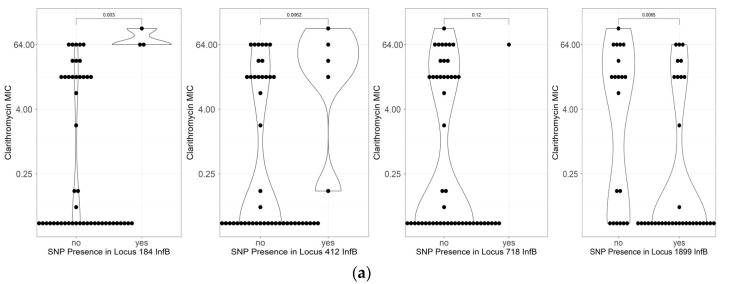

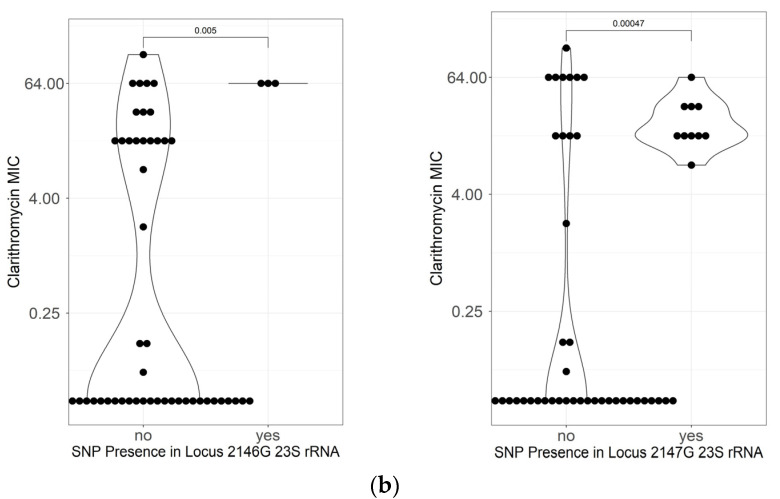
Distributions of MIC in the presence and absence of mutations. The MIC is plotted as *y*-axis and mutation were represented by dots. Mutations in *infB* and 23S rRNA genes associated with higher clarithromycin MIC. (**a**) MIC comparisons mutations in the *infB genes*. (**b**) MIC comparisons mutations in the 23S rRNA *genes*.

A mutation in locus 718 in the *infB* gene also been reported to be associated with resistant isolates; although, they were also found in the susceptible isolates. Isolates possessing mutation in A412G also showed high MIC (Figure 4). Only alteration in locus 184 showed extremely high MIC (64–128 mg/L). All of these mutations were found together with mutations on 23S rRNA. Therefore, it did not improve the agreement between phenotype and genotype.

## 3. Discussion

*H. pylori* antimicrobial resistance remains a severe problem in *H. pylori* eradication. Mutations in the target genes have been proposed to be the most common mechanism in resistance of *H. pylori.* Therefore, we examined known mutations and SNPs in antibiotic protein targets using this dataset. In South Asia, both a high prevalence of infection and a high prevalence of antibiotic resistance have been recorded. A high prevalence of levofloxacin and metronidazole-resistant isolates was also reported in the other parts of India, 65.6% and 81.2%, respectively [16].

The genomic diversity of *H. pylori* varied according to geographic location [14]. These genetic variations also occurred, *gyrA* and *gyrB* [8]. This observation is consistent with this dataset, which contained a high prevalence of levofloxacin resistance. The *gyrA* mutations D91N and N87K were more frequent in the cluster that shared a branch with hpEurope than the ones with hpAsia2. N87K and D91N mutations were located in the Quinolone Resistance Determining Region (QRDR) of the *gyrA H. pylori* [17]. The mutations in this region were found in the resistant isolates in many previous studies [18,19,20]. Interestingly, one strain containing a mutation in both loci (87 and 91) showed high-level resistance. As described in a previous study, isolates from patients who failed eradication by sitafloxacin also showed high MIC and double-mutation mutations that occurred in more than one locus [21]. Complementation of both mutations that worsen the stability of the protein could be a plausible explanation.

Amoxicillin resistance is included in the first-line regiments of *H. pylori* eradication [4]. Amoxicillin resistance is the result of a mutation in the penicillin-binding motif of Penicillin Binding Protein (Pbp). SNPs in the *pbp1a*, *pbp2*, *pbp3*, and *pbp4* genes were investigated. We discovered the well-known N562Y mutation in the *pbp1a* gene and the most prevalent mutations seen in *H. pylori* amoxicillin-resistant isolates. The natural transformation also demonstrated the crucial role of mutations to increase the minimum inhibitory concentration levels [11]. It concerns the motif SNN559-561, one of the three *pbp1A* motifs defining the active site of the enzymes [22]. These motifs are located at the SAIK368-371 motif and have already been reported before in association with amoxicillin resistance. The others (K306R, T30S, and V45I) turn out to be new candidates except for V45I, which has been already reported in previous studies [9,23]. Therefore, further investigation in vitro is needed to establish their association with the resistance. Nevertheless, these loci do not fall among the binding sites and V45I is present in 11.1% of susceptible isolates. Mutations in *pbp2*, *pbp3*, and *pbp4* were also found among the significant mutations. However, they were rarely found in both isolates. Perhaps, the statistical significance might be due to this unbalanced sampling. In addition, the effect of mutation accumulation is hard to determine since both isolates had MIC values of 0.25 mg/L. Further explorations would shed more light on these mutations.

Various alterations have been described up to this point, but they are inconclusive due to the global spread of metronidazole resistance [24,25]. In fact, just three isolates in this dataset were sensitive to metronidazole. High-resistance NADPH reductase encoded by the *rdxA* gene is the target of metronidazole. In this study, isolates possessing frameshift mutations in *rdxA* were resistant. Frameshift mutations were also prevalent in the *frxA* genes among resistant isolates; although, they were also present in the susceptible isolates. The analysis also expanded to the ferric uptake regulator (fur), a modulator of drug activity B (*mdaB*), riboflavin biosynthesis protein (*ribF*), and *omp11* [7]. A previous study reported mutations in loci 222 and 227 of *ribF*, but none of them was found in ours [26]. Instead, significant associations with the very high metronidazole MIC were found in loci D253E and S151L of *ribF.* The afore-mentioned study also found a mutation in locus 114, whereas the current one showed significance in locus N118Q *fur.* The stability index analysis showed that the mutation affects the stability index; although, further docking analysis with metronidazole and experimental analysis is necessary. These results also indicate that multiple mechanisms might be involved in metronidazole resistance.

Clarithromycin suppresses protein synthesis by binding to the peptidyl-transferase of the 23S ribosomal RNA (rRNA). In the clarithromycin resistance isolates, the mutations decreased. The A2142G mutation was more prevalent than the A2143G mutation [27,28]. Resistance to metronidazole and clarithromycin was observed in 41.6% of isolates. Mutations at locus 2146 and 2147 of 23S rRNA (formerly known as locus 2142 and 2143) account for most of the clarithromycin resistance. The mutations in these two loci were only found in the resistant isolates. According to prior research in South Asia, the substitution of adenine for guanine at locus 2147 is more prevalent than the substitution of adenine for cytosine [8]. The mutations in 2147 are also more prevalent than those in locus 2146, which is coherent with the previous study [27]. Unlike previously reported studies in Bangladesh [29], the mutation related to clarithromycin resistance could be influenced by the genome population, as has been shown in most cases (90%), the A2147G mutation belonged to hpEurope. Further study using the worldwide dataset is necessary to confirm the results.

However, the mutations in loci 2146 and 2147 were only present in 50% of isolates. Previous studies reported the mutation in another locus of 23S rRNA, such as 2182 [30]. However, there were no significantly associated SNPs found in our dataset. Therefore, we searched the SNPs in the genes previously reported by our group to induce clarithromycin resistance: *infB.* The *infB* gene encodes the translation initiation factor IF-2, which stimulates the binding of formyl methionyl-transfer RNA to 30S ribosomal subunits during the initiation of protein synthesis and is implicated in the hydrolysis of GTP during the development of the 70S ribosomal complex. Although we could not find the mutation in a similar location (locus 160), we discovered that the alteration of cytosine to thymine in locus 718 was significantly associated with resistance, as well as in locus 184 and 412, which could be the new candidates for further investigation. These mutations were first reported in this study, and they were located in four separate locations. Further evaluation on finding the binding motifs is necessary.

Conducting an AMR test prior to the treatment also increases the eradication efficacy [31]. Genotypically guided treatment, especially for clarithromycin and levofloxacin, was also reported to be effective [32]. The findings in this study with the well-known mutations in *gyrA* and 23SrRNA support the utilization of genotypic-guided therapy. Genotypic-based results are faster to obtain compared to phenotypic. Previous studies detected resistant *H. pylori* in clinical specimens with excellent specificity and sensitivity using more straightforward techniques such as restriction fragment length polymorphism (RFLP), 3′-mismatch PCR, DNA sequencing, the PCR line probe assay (PCR-LiPA), and fluorescence in situ hybridization (FISH) [33]. Nonetheless, in order to acquire a greater resolution and investigate mutations not previously identified, whole genome sequencing analysis is required. The mutations found in the current study utilized the ARIBA pipeline to identify the mutations found in the current study and showed similar results to our previous findings, indicating that this method could be an alternative to use directly from the FASTQ file without going through genome assembly [24]. This study’s limitations include the number of datasets used. In addition, the possibility of recombination and linkage between loci in the genomes of bacteria was not considered. Therefore, it is necessary to conduct additional research with a larger dataset that has been meticulously curated to determine the genetic population.

## 4. Materials and Methods

### 4.1. Patient Sampling and H. pylori Isolates

The *H. pylori* isolates used as the primary dataset in this study were obtained from gastric biopsies of 133 patients during a survey conducted in 2014 in Dhaka Medical College Hospital, Bangladesh. Each subject was given the information for consent and submitted the informed consent. To obtain a single colony of *H. pylori* isolates, biopsy specimens from the antrum were homogenized in phosphate-buffered saline (PBS), inoculated in *H. pylori* selective plates (*Nissui* Pharmaceuticals Co., Tokyo, Japan), and incubated for five days in microaerophilic environment (5% O_2_ and 10% CO_2_). Before harvesting for genomic DNA extraction, each colony was subcultured on Brucella agar (BD BBL, Becton Dickinson and Company, Franklin Lakes, NJ, USA) supplemented with 7% horse blood. *H. pylori* were isolated from 56 patients suffering from chronic gastritis (53/56) and peptic ulcers (3/56). This study’s protocol was approved by the Oita University Faculty of Medicine in Japan and the Ethics Committee of the Bangladesh Medical Research Council (BMRC) in Dhaka, Bangladesh.

### 4.2. Antibiotics Susceptibility Test

All isolates were tested for susceptibility to five antibiotics by Etest (Biomerieux, Nice, France) [15]. The bacteria were subcultured three times from the −80 °C bacterial stock on Brucella agar plates with 7% horse blood and no antibiotics in microaerophilic conditions. Day 2 culture of *H. pylori* was suspended in PBS and modified its bacterial density to 0.5 Mc Farland standard. *H. pylori* were inoculated onto Mueller–Hinton agar ((BD BBL, Becton Dickinson and Company, Franklin Lakes, NJ, USA) with 10% horse serum, and an Etest strip was inserted in the middle according to manufacturer’s instructions. Clarithromycin, amoxicillin, tetracycline, and metronidazole ranged from 0.016 mg/L to 256 mg/L, whereas levofloxacin ranged from 0.002 to 32 mg/L. After 72 h of microaerophilic incubation, the results were evaluated. We also calculated the MIC50 as the median to show the antibiotic’s ability to inhibit 50% of isolates. The European Committee on Antimicrobial Susceptibility Testing (EUCAST) clinical breakpoints for antibiotic susceptibility tests were amoxicillin 0.125 mg/L, clarithromycin 0.5 mg/L, levofloxacin 1 mg/L, metronidazole 8 mg/L, and tetracycline 1 mg/L (EUCAST, 2022; available at https://www.eucast.org/clinical_breakpoints (accessed on 10 May 2022). 

### 4.3. Genome Sequencing

*H. pylori* were harvested in PBS and DNA was extracted using the Qiagen DNEeasy Kit, per the manufacturer’s instructions (Hilden, Germany). The Quantifluor dsDNA System (Madison, WI, USA) and Quantus Fluorometer measured DNA concentration (Sunnyvale, CA, USA). After standardizing the concentration, MiSeq Illumina whole-genome sequencing produced 300 bp paired-end reads. We constructed a full genome alignment of all Bangladesh sequences and a prior study that defined population to analyze genetic relatedness and population genetics [34]. Snippy-core version 4.6.2 (available at https://github.com/tseemann/snippy (accessed on 18 April 2022)) aligned was used to align the genome, FastTree 2.0 with GTR–nt mode was utilized to create a maximum probability tree from the alignment [35], and Microreact was used to visualize the tree [36].

### 4.4. Analysis of the SNP and Protein Modelling

ARIBA pipeline analyzed the SNPs. A reference database was created by collecting antibiotic-related genes of strain 26,695. Coding sequences and novel variants contained ARIBA metadata and CD-HIT clusters’ references. The 56 isolates’ fastq readings were mapped to the cluster’s reference sequence and assembled individually. Bowtie mapped reads to cluster references, then SAMtools called variations. Strain results included assembled genes and variations compared to strain 26,695. Text files summarized the reports. If coverage and identity exceeded 50% and 90%, the genes were present. SNPs with amino acid variations were summarized, and those that occurred in fewer than 10% of isolates were eliminated. Antibiotic resistance phenotype was correlated with SNP occurrence and absence. Fisher’s exact test *p* < 0.05 was significant. Spearman’s rank correlation analyzed the other non-parametric correlation. R was used for statistical analysis and graphing (version 3.5.1).

To assess the role of mutation in RibF and the Fur gene, a protein model was constructed from the genes of 26,695 using Phyre2 [37]. The physicochemical characteristics were evaluated using Expasy [38] and compared between the wild-type isolates and mutant isolates.

## 5. Conclusions

Significant correlations exist between genetic *H. pylori* variables discovered by WGS and phenotypic resistance. This enables the prediction of phenotypic clarithromycin, amoxicillin, metronidazole, and levofloxacin resistance in a clinically relevant timeframe based on genotypic information in the 23S rRNA *infB*, *pbp1a*, *gyrA*, *gyrB*, and *rdxA*, genes with a substantial concordant. This result may facilitate the rapid determination of drug susceptibility in personalizing treatments to increase the efficacy of *H. pylori* eradication.

## Figures and Tables

**Table 1:** Mutations associated with levofloxacin resistance in *H. pylori*.

SNP	Resistant (n = 37)		Sensitive (n = 19)		Total	*p*-Value
	Absent	Present	Absent	Present		
*gyrA*						
D91G	32	5	19	0	56	0.15
D91N	24	13	19	0	56	0.002
D91Y	36	1	19	0	56	1
N87K	24	13	19	0	56	0.002
N87Y	36	1	19	0	56	1
*gyrB*						
A343V	29	8	19	0	56	0.041

*p*-Values < 0.05 were determined as significant association.

**Table 2:** Significant mutations found in Amoxicillin resistance in *H. pylori*.

Genes	AMX Resistant (n = 2)		AMX Susceptible (n = 54)		*p*-Value
	Present	Absent	Present	Absent	
*pbp1a*					
T30S	1	1	0	54	0.0357
V45I	2	0	6	48	0.0182
K306R	1	1	0	54	0.0357
V374L	1	1	0	54	0.0357
N562Y	2	0	0	54	0.0006
G594fs	1	1	0	54	0.0357
*pbp2*					
S79A	1	1	0	54	0.0357
F99S	1	1	0	54	0.0357
E312G	1	1	0	54	0.0357
G529V	1	1	0	54	0.0357
P582T	1	1	0	54	0.0357
*pbp3*					
I14V	2	0	4	50	0.0097
I191V	2	0	8	46	0.0292
V223M	2	0	7	47	0.0234
*pbp4*					
Q226R	1	1	0	54	0.0357
T272A	1	1	0	54	0.0357

*p*-Values < 0.05 were determined as significant association.

**Table 3:** Genetic variations associated with metronidazole resistance in *H. pylori*.

Genes	Frameshift	Truncated	SNP	Resistant		Susceptible	
				n Total	Average	n Total	Average
*ribF*	2	0	67	615	11.6	38	12.7
*frxA*	14	5	81	231	4.4	14	4.7
*rdxA*	8	5	95	353	6.7	14	4.7
*mdaB*	10	1	40	289	5.5	11	3.7
*omp11*	1	0	17	52	1.0	0	0.0
*fur*	0	0	14	42	0.8	2	0.7

**Table 4:** The point mutation occurred in the clarithromycin resistance.

Row Labels	Resistant (n = 22)		Susceptible (n = 34)		*p*-Value
	Yes	Percentage	Yes	Percentage	
23S rRNA					
A2146C	1	4.5%	0	0.0%	0.39
A2146G	3	13.6%	0	0.0%	0.05
A2147G	10	45.5%	0	0.0%	1.8 × 10^−5^
*infB*					
C718T	8	36.4%	3	8.8%	0.024239
A1899G	10	45.5%	26	76.5%	0.016799

## Data Availability

All genome data were stored in Genbank with BioProject accession PRJDB11821.

## Supplementary Materials

The following supporting information can be downloaded at https://www.mdpi.com/article/10.3390/antibiotics12020279/s1, Table S1. The distribution of mutations in the *gyrA* gene between the *H. pylori* population; Table S2. Frameshift mutations occurred in the *rdxA*, *mdaB*, *ribF,* and *frxA;* Table S3. Physical and chemical parameters of the Fur and RibF, Figure S1. The protein structure prediction of RibF and Fur.
