# Supplementary material for: Mutations Related to Antibiotics Resistance in Helicobacter pylori Clinical Isolates from Bangladesh
Source: Antibiotics (Basel). 2023 Jan 31;12(2):279. doi: 10.3390/antibiotics12020279 (PMC9952455; doi:10.3390/antibiotics12020279)
Supplement: Supplementary file 1 [file antibiotics-12-00279-s001.zip › antibiotics-2173447-supplementary.pdf]

Supplementary Table S1. The distribution of the mutation in *gyrA* among hpAsia2 and hpEurope

Mutations	Resistant			Susceptible			Grand Total
	hpAsia2	hpEurope	Total	hpAsia2	hpEurope	Total	
N87K	5	8	13	0	0	0	13
N87Y	1		1	0	0	0	1
D91G	3	2	5	0	0	0	5
D91N	3	10	13	0	0	0	13
D91Y	1		1	0	0	0	1
Total	13	20	33	0	0	0	

Supplementary Table S2. Frameshift mutations occurred in the *rdxA, mdaB, ribF and frxA*

	Resistant		Susceptible		
Framehift locus	Absent	Present	Absent	Present	Grand Total
rdxA.H97fs	46	7	3		56
rdxA.I160fs	52	1	3		56
rdxA.K64fs	52	1	3		56
rdxA.K78fs	52	1	3		56
rdxA.K8fs	52	1	3		56
rdxA.M84fs	51	2	3		56
rdxA.P96fs	20	33	2	1	56
rdxA.R41fs	52	1	3		56
mdaB.A51fs	36	17	2	1	56
mdaB.E55fs	38	15	3		56
mdaB.G159fs	52	1	3		56
mdaB.H50fs	15	38	1	2	56
mdaB.P68fs	53		2	1	56
mdaB.S87fs	47	6	3		56
mdaB.V171fs	52	1	3		56
mdaB.V44fs	52	1	3		56
mdaB.V57fs	39	14	3		56
ribF.R256fs	52	1	3		56
ribF.S258fs	52	1	3		56
frxA.A16fs	51	2	3		56
frxA.D109fs	52	1	3		56
frxA.D136fs	52	1	3		56
frxA.D94fs	51	2	3		56
frxA.G69fs	51	2	3		56
frxA.K105fs	52	1	3		56
frxA.K18fs	45	8	2	1	56
frxA.K204fs	52	1	3		56
frxA.K97fs	52	1	3		56
frxA.M66fs	52	1	3		56
frxA.Q10fs	52	1	3		56
frxA.S189fs	53		2	1	56
frxA.V36fs	52	1	3		56
frxA.W68fs	51	2	3		56
Grand Total	1583	166	92	7	1848

Supplementary Table S3. Physical and chemical parameters of the fur and ribF protein

**Protein**	**Theoretical pI**	**Estimated half-life (mammalian reticulocytes, in vitro; yeast, in vivo; Escherichia coli, in vivo)**	**Instability index**	**Aliphatic index**	**Grand average of hydropathicity (GRAVY)**
fur_WT	8.50	30hr; >20hr; >10hr	54.19	85.73	-0.631
fur_N118Q	8.50	30hr; >20hr; >10hr	55.47	85.73	-0.631
ribF_WT	9.33	5.5h; 3min; 2min	36.25	111.11	-0.148
ribF_D253E	9.33	5.5h; 3min; 2min	36.25	111.11	-0.148
ribF_S151L	9.33	5.5h; 3min; 2min	36.25	112.51	-0.131

Supplementary information:

A.

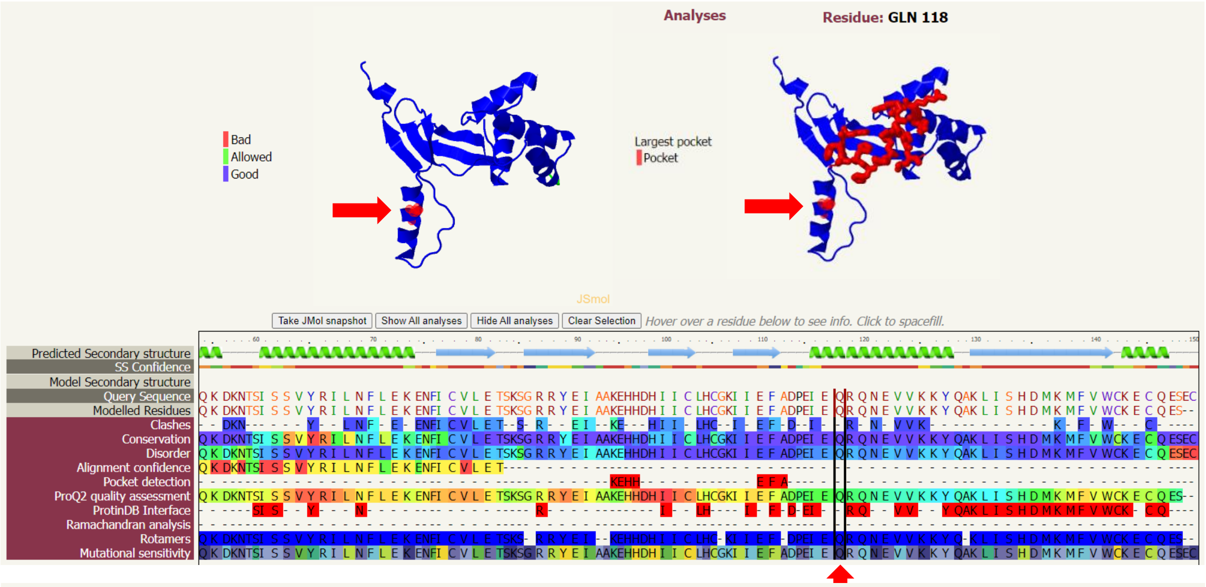

B.

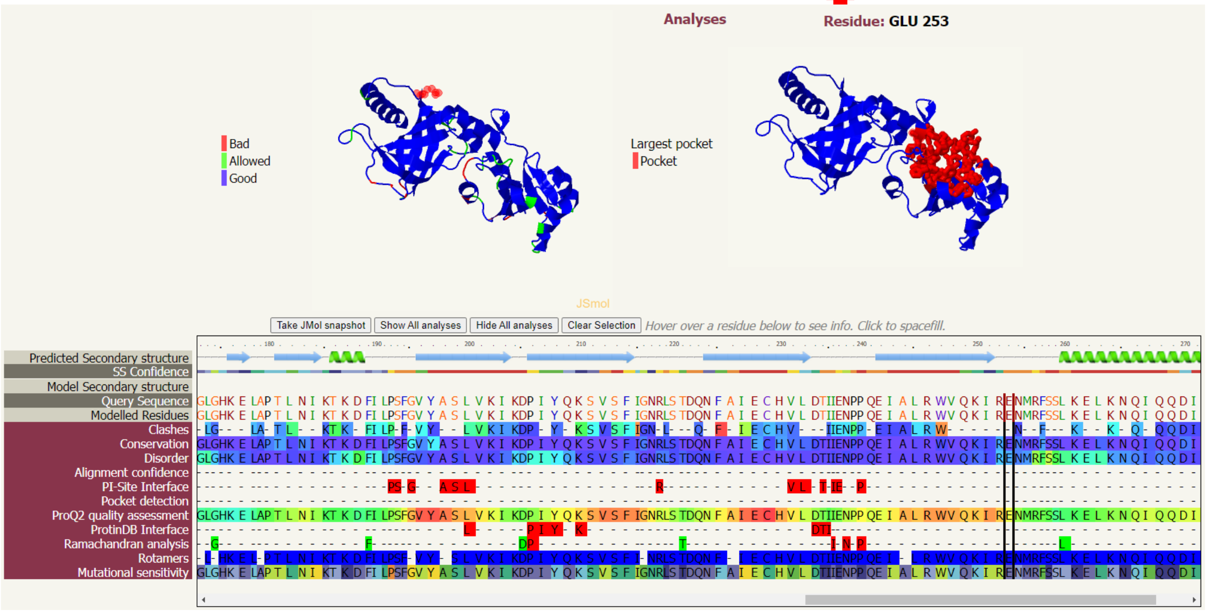

C.

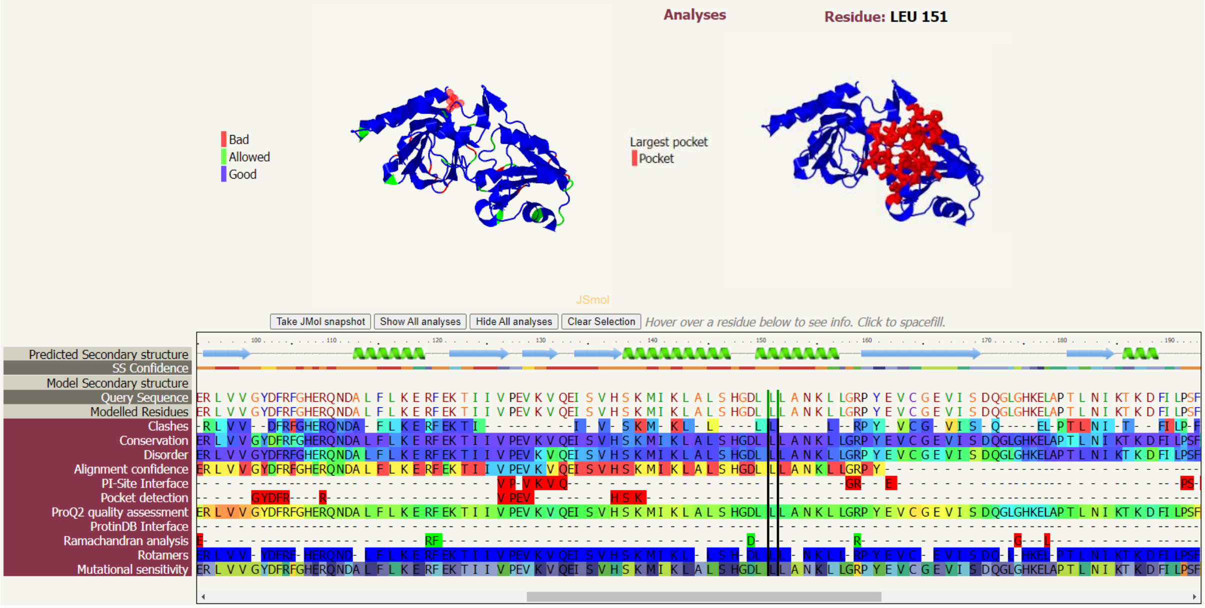

Supplementary Figure S1. A. In-depth analysis on fur_N118Q amino acid. B. In-depth analysis on ribF_D253E amino acid. C. In-depth analysis on ribF_S151L amino acid. *Red arrow*: represents the location of mutation. *Left structure*: the colour represent the Ramachandran analysis results. *Right structure*: the red mark showed the pocket that possibly become the protein active site. *Bottom table*: informed the secondary structure and other protein analysis results.
